# Effect of Terroir on Phenolic Content and Aroma Properties of Grapes and Wines

**DOI:** 10.3390/foods14081409

**Published:** 2025-04-18

**Authors:** Yuyuen Phajon, Hongbing Tan, Bochen Liu, Yang Zhang, Yanlun Ju, Tian Shen, Meilong Xu, Yulin Fang

**Affiliations:** 1 College of Enology, Northwest A&F University, Yangling 712100, China; phajon@hotmail.com (Y.P.); 2023056361@nwafu.edu.cn (H.T.); lbc@nwsuaf.edu.cn (B.L.); 2020056205@nwafu.edu.cn (Y.Z.); juyanlun2016@nwsuaf.edu.cn (Y.J.); 2 Horticultural Research Institute, Ningxia Academy of Agriculture and Forestry Sciences, Yinchuan 750002, China; st_910101@163.comst (T.S.); xml1106007@163.com (M.X.); 3 Shaanxi Engineering Research Center for Viti-Viniculture, Yangling 712100, China; 4 Heyang Viti-Viniculture Station, Northwest A&F University, Yangling 715300, China

**Keywords:** terroir, vineyards, wine regions, phenolic compounds, volatile compounds

## Abstract

Ten vineyards, belonging to five wine regions, were selected in this study. Maturity indicators and phenolic and aromatic compounds were measured to investigate relationships with factors related to the terroir, including climate conditions and soil nutrients. Multiple factor analysis of all compositions showed that different wine regions or vineyards had different characteristics, which were partly associated with the terroir. The results showed that Shizuishan had high sugar content. A high level of anthocyanins could be found in Qingtongxia and Yongning, respectively. Moreover, Qingtongxia had higher concentrations of monomeric phenols in grapes than the others. LSYH (Lanshanyunhao) and LL (Lilan) had higher YAN (yeast assimilable nitrogen) content and pH in grapes, but their wine had a green flavor (high concentration of alcoholic volatile compounds). The Shizuishan and Hongsipu wine regions had fruitier flavors (high concentration of ester volatile compounds). This study demonstrated the characteristics of different vineyards and wine regions, providing a direction for the future development of region-specific grapes and wines.

## 1. Introduction

The eastern foothills of the Helan Mountains in Ningxia Autonomous Region, China, is a moderate and temperate arid climate zone with excellent natural resources for the cultivation of wine grapes and the development of the winemaking industry; Cabernet Sauvignon is the main grape variety planted in Ningxia [1,2]. Based on the effective cumulative temperature data, Ningxia is divided into five sub-regions: Shizuishan, Yinchuan, Yongning, Qingtongxia, and Hongsipu. Therefore, each sub-region has a different climate. One of the most important elements influencing grapes and wine is the terroir. The terroir includes the interaction between soil, topography, and geology and the interaction between the macroclimate and mesoclimate [3,4].

The differences in the characteristics of grapes and wines from different wine regions are reflected in ripeness indicators, phenolic variations, and differences between aromatic substances. Therefore, the differences between these substances can further distinguish the characteristics of the grapes and the wines from one wine region to another.

The terroir exerts different effects on ripeness indicators, phenolic variations, and aromatic substances. As previous studies have pointed out, climatic factors, especially light and temperature, play a significant role in the accumulation of glucose during the period from veraison to ripening; soil factors, particularly nitrogen content, also have a significant impact on quality [5]. In terms of phenolics, tannins and anthocyanins are the most abundant substances, which determine the appearance of the wine regarding color, taste, and antioxidant activity [6,7]. Climate factors also have a significant effect on the accumulation of phenolics. For example, anthocyanin content is regulated by light and temperature [8]. Slightly alkaline to neutral pH soil makes it easier for plants to absorb nutrition, improving their growth and fruit quality [1]. Soil nutrition can impact the sugar and tannin content of grapes [1]. Aromas are affected by both climate and soil, which are essential factors that determine the nature and quality of wines [9,10]. Therefore, the analysis of wine aromas among different wine regions is far-reaching for characterizing wine regions and vineyards. As an emerging producing area, the influence of the terroir of the eastern Helan Mountains on the factors determining the quality of grapes and wines is still unclear. Moreover, the whole region still faces many problems, such as the lack of attention to the quality of wine grapes in different wine regions, which leads to the uneven quality of grapes in each area and the lack of characteristics in the wine-producing areas.

In this study, the quality of grapes and wines from different wine regions and vineyards was analyzed by revealing the relationship between the terroir and sugar, as well as acid and phenolic and aromatic substances. This will enable the development of a grape and wine industry with local characteristics in the future in different wine regions.

## 2. Materials and Methods

### 2.1. Field Conditions and Materials

The original ‘Cabernet Sauvignon’ grape berries were collected from five wine regions (ten commercial vineyards) in Ningxia. The specific geographical information is as follows: Shuizuishan: XYWQ (Xiyuwangquan vineyard) at 39°134′ N, 106°566′ E and HD1 (Hedong vineyard) at 38°989′ N, 106°320′ E; Yinchuan: LSYH (Lanshanyunhao vineyard) at 38°738′ N, 106°104′ E and HJZ (Heijinzun vineyard) at 38°733′ N, 106°083′ E; Yongning: LL (Lilan vineyard) at 38°284′ N, 105°984′ E and XXW (Xixiawang vineyard) at 38°257′ N, 106°041′ E; Qingtongxia: YM (Yuma vineyard) at 38°090′ N, 105°918′ E and XG (Xige vineyard) at 38°076′ N, 105°886′ E; Hongsipu: LHY (Longhuiyuan vineyard) at 37°328′ N 106°175′ E and HD2 (Huida vineyard) at 37°472′ N, 106°093′ E.

At each vineyard, 30–50 grape plants with similar growth and the same frame shape and that were free of pests and diseases were randomly selected. About 200–300 grapes of similar size were sampled and harvested for further analyses when the soluble solid content reached 23–25 °Brix.

### 2.2. Vinification

About 20 kg of grapes were sorted, destemmed, crushed, and placed in 30 L stainless steel containers; then, 50 mg/L of SO_2_ and 30 mg/L of pectinase (Lallzyme Ex) were added and manually mixed. The must was macerated for 24 h, and then 0.2 g/L of active dry yeast (*Saccharomyces cerevisiae* strain, Lallemand, Danstar Ferment AG, Zug, Switzerland) was added. Fermentation was carried out at room temperature, 20 °C, with pressurization and stirring three times a day, and finished to dryness (reducing sugar < 4 g/L). Temperature and density controls were maintained during this process. After fermentation, wine samples were bottled and stored at 4 °C for further analyses.

### 2.3. Determination of Soil Samples and Microenvironmental Indicators

#### 2.3.1. Soil Collection

For each vineyard, ten sampling points were selected according to the principle of random equal volume using the S-shaped distribution method. Soil samples were collected at three depths of 0–20 cm, 20–40 cm, and 40–60 cm with a soil drill. Three soil samples from the same depth at each sampling point were placed and mixed on clean woven bags, and then some soil samples were collected in bags. After the samples were transported back to the laboratory, small portions of the soil samples were air-dried.

#### 2.3.2. Determination

Organic matter: potassium dichromate volumetric method [11]. pH: acidity meter method [12]. fast-acting nitrogen: potassium chloride leaching and rapid distillation method [13]. fast-acting phosphorus: molybdenum antimony anti-colorimetric method [14]. fast-acting potassium: ammonium acetate extraction method [15].

Temperature and humidity were measured using a temperature and humidity recorder (RC-4, Jiangsu Jingchuang Company, Xuzhou, China) from 5th July to the time of harvest.

The data were collected every hour and obtained after the grapes were ripe.

The measurement of light intensity and spectral distribution was carried out (FX2000, Shanghai Fuxiang Company, Shanghai, China) between 11:00 a.m. and 1:00 p.m. on a sunny day, once every 7 days, with 4 measurement points per vineyard, taking into account both the shade and the sun, and the average value was taken as the final measurement results.

### 2.4. Physicochemical Parameters of Grapes

#### 2.4.1. Sugar and Acid Contents

The titratable acidity (TA) (g/L tartaric acid) was measured according to the OIV method [16]. The pH was detected by a digital pH meter (PB-10; Sartorius, Göttingen, Germany). And a digital refractometer (PAL-1; Atago Co., Ltd., Tokyo, Japan) was used to determine the total soluble solid (TSS) content and expressed it as °Brix.

#### 2.4.2. Extraction of Phenolic Compounds

The extraction and measurement of phenolics from grape skins were performed with reference to [17]. using a methanolic solution of hydrochloric acid, and the extracts were stored at −20 °C; wine samples were used directly for analysis.

#### 2.4.3. Determination of Phenolic Compounds

Total phenols: The total phenolic content of grape berries and wine was determined using the Folin–Ciocalteu method, and the results were expressed as gallic acid equivalents [18]. Total anthocyanins: The total anthocyanin contents of grape skins and wine were determined using the pH differential method, and the results were expressed as cornetin-3-glucoside equivalents [19]. Total flavan-3-ol: The measurement of the total flavan-3-ol in grape skin was performed using the p-DMACA-hydrochloric acid method. The results were expressed as (+)-catechin equivalents [20]. Total flavonoids: The determination of total flavonoids in grapes and wine was performed by using the p-dimethy-laminocinnamaldehyde-HCl method. The results were expressed as rutin equivalents [21].

### 2.5. Monomeric Sugar

#### 2.5.1. Extraction of Samples

The extraction was conducted according to the method of [17] with slight modifications. Briefly, 0.3 g of grapes was added to 10 mL of ultrapure water and centrifuged. The supernatant was bottled in 50 mL volumetric flasks, and the precipitate was washed twice with ultrapure water. The supernatant was combined and fixed the volume to 50 mL, and then it was passed through an aqueous 0.22 μm filter membrane for ion chromatography with the electrochemical potential detector.

#### 2.5.2. Determination of Samples

The chromatographic conditions were established as follows: The separation was performed using a CarboPac PA 10 column (4 mm × 250 mm), while a PG 10 guard column (4 mm × 50 mm) was employed for protection, with the mobile phase consisting of 3 mmol NaOH delivered at a flow rate of 0.8 mL/min. The system operated under controlled parameters where the column temperature was maintained at 28 °C, and an injection volume of 25 μL was consistently applied.

### 2.6. Organic Acid

#### 2.6.1. Extraction of Samples

The grapes were squeezed and centrifuged at 5000× *g*. The 5 times diluted supernatant was aspirated at 1 mL and filtered through a 0.22 μm microporous membrane.

#### 2.6.2. Determination of Samples

The waters 1525 HPLC system was used with a DIKMA Platisil NH2 (250 × 4.6 mm, 5 μm) column with the following conditions: mobile phase 25 mM potassium dihydrogen phosphate (pH adjusted to 2.5 by phosphoric acid), flow rate 1.0 mL/min, column temperature 25 °C, waters 2998 diode array detector, detection wavelength 210 nm. The detection wavelength was 210 nm.

### 2.7. Monomeric Anthocyanins

#### 2.7.1. Extraction of Grape Skins

Monomeric anthocyanin extraction of grape skins was performed by referring to the method of [22]. An Agilent 1100 series LC/MSD Trap-VL liquid chromatograph ion trap mass spectrometer with a diode array detector (DAD) was used for qualitative and quantitative analysis of the samples.

#### 2.7.2. Determination

Samples for grapes and wine were filtered through 0.45 µm inorganic filter membranes (polyethersulfone), and samples were directly analyzed by HPLC-MS. The chromatographic conditions were as follows: mobile phase A: formic acid–acetonitrile–water (2:6:92, V:V:V), mobile phase B: formic acid–acetonitrile–water (2:54:44, V:V:V). The mobile phase elution procedures were as follows: 1~18 min, 10~25% B; 18–20 min, 25% B; 20~30 min, 25~40% B; 30~35 min, 40~70% B; 35~40 min, 70~100% B. The mobile phase flow rate was 1.0 mL/min, the column temperature was 50 °C, the detection wavelength was 525 nm, the wavelength scan was 200–900 nm, and the injection volume was 30 µL. The mass spectrometry conditions, with an electrospray ionization source (ESI) and positive ion mode, were as follows: the ion scan range was 100~1500 *m*/*z*; the nebulizer pressure was 35 psi; the drying gas flow rate was 12 L/min; and the drying gas temperature was 300 °C.

### 2.8. Monomeric Phenols

#### 2.8.1. Extraction of Monomeric Phenols from Grape Skins

Place 1 g of dried grape skin powder in a 50 mL centrifuge tube and add 1 mL of distilled water and 9 mL of ethyl acetate. The solute was shaken for 30 min at 25 °C (130 rpm) and centrifuged at 8000 rpm for 5 min. Then, the supernatant was transferred to a 50 mL centrifuge tube with a 1 mL pipette. The process was repeated 4 times (adding a black bag to shade the supernatant). The supernatant (total 40 mL) was combined and evaporated to dryness at 33 °C on a rotary evaporator, and the chromatographic methanol was fixed to 1 mL.

#### 2.8.2. Extraction of Monomeric Phenols from Wine

Using a thin film rotary evaporator (<40 °C), 30 mL of the organic phase is concentrated to dryness, and the residue is dissolved in 3 mL of chromatographic methanol and stored at −20 °C, protected from light, for liquid phase analysis. The sample was filtered through a 0.45 μm microporous membrane prior to measurement.

#### 2.8.3. Qualitative and Quantitative Analysis

The quantitative analysis was performed according to the standard curve of the standard sample. The chromatographic conditions were as follows: injection volume of 10 μL; Agilent ZORBAX SB-C18 column (250 mm × 4.6 mm × 5 μm); mobile phase A: 2% glacial acetic acid aqueous solution; mobile phase B: chromatographic acetonitrile; detection wavelength of 280 nm; flow rate of 1 mL/min; column temperature of 30 °C. The elution program was as follows: 0~20 min, B is 3~5%; 20~35 min, B is 5~15%; 35~50 min, B is 15~30%; 50~65 min, B is 30~30%; 65~75 min, B is 30~0%.

Mixed standard solutions of monomeric phenolics at five different concentration levels were prepared, with each level replicated three times. Standard curves were established using the average peak areas corresponding to each concentration (mg·L⁻^1^). The identification of monomeric phenolics was based on retention times, and the concentrations of these compounds in the samples were calculated using the external standard method.

### 2.9. Volatile Compounds

#### 2.9.1. Extraction of Volatile Compounds

HS-SPME-GC-MS (headspace solid-phase microextraction coupled with gas chromatography) was used to determine the aroma substances. In total, 50 g of grapes stored at −80 °C were frozen in liquid nitrogen, deseeded, and destemmed. Then, 1 g PVPP and 0.5 g D-gluconolactone were added and crushed to powder form; the grapes were macerated at 4 °C for 120 min; the juice was rapidly centrifuged at 4 °C and 2000× *g* for 10 min; and the clarified grape juice was collected. In total, 5 mL of the above supernatant was taken in a 15 mL sample bottle, and 1 g NaCl, 10 μL of 2019 μg/L internal standard 4-methyl-2-pentanol, and a magnetic rotor were added; then, it was placed into a sample bottle with a cap on a solid-phase microextraction bench. The supplant was equilibrated at 40 °C, 500 r/min for 30 min. The activated extraction head was inserted into the top of the sample extraction bottle, and then the sorbent head was exposed to the headspace vapor of the extraction bottle. The sample was extracted at 40 °C for 30 min, and the extraction head was removed and inserted into the GC-MS inlet and thermally resolved at 250 °C for 8 min.

#### 2.9.2. Qualitative and Quantitative Analysis

The chromatographic column was a DB-wax column (30 m × 250 μm × 0.25 μm). The carrier gas was high-purity helium at a flow rate of 1 mL/min. The inlet temperature was 250 °C, and the resolution time was 8 min in non-split injection mode. The mass spectrometry interface temperature was 280 °C, and the mass scan range was 30–350 u.

The aroma profile was analyzed by GC-MS analysis software (MassHunter Workstation, 12.1), and the samples were characterized by comparison with the retention time, retention index, and mass spectral information of the standards under the same chromatographic conditions, and semi-quantitative analysis was performed by the internal standard method.

Semi-quantitative analysis refers to the estimation of the approximate concentration range of a volatile substance from relative peak areas rather than the precise determination of the absolute content. It does not require a standard curve and does not rely on a pure standard substance of the target compound to establish a calibration curve [23].

### 2.10. Statistical Analysis

ArcGIS10.8 was used to visualize available nitrogen, potassium, and phosphorus. Statistical analysis of all data was performed using SPSS 24.0 (IBM, Armonk, NY, USA). One-way ANOVA and Duncan’s multiple polar difference tests were used for statistical testing (*p* < 0.05). Multiple factor analysis (MFA) was used to represent relationships across data. All statistical analyses were conducted using R version 3.6.0.

## 3. Results and Discussion

### 3.1. Analysis of Ecological Factors in Different Production Areas

#### 3.1.1. Climate Conditions

Ecological factors play important roles in the quality of both grapes and wine. This study compared rainfall, average humidity, average temperature, and net light intensity in ten vineyards from July to September. The data on rainfall can be seen in Table 1. Rainfall in the Shizuishan wine region and the Yongning wine region was less than 30 mm. In the rest of the regions, Yingchuan, Qingtongxia, and Hongsipu were characterized by more than 32 mm of rainfall, with the highest one (33.09 mm) found in the Yingchuan region during the same period.

As shown in Table 1, starting from the Yinchuan wine region, the average temperature decreased as the geographical location moved south. LSYH vineyard had the highest average temperature (27.4 °C), followed by HJZ and LL vineyards (both 26.5 °C). The lowest average temperature could be seen in the LHY vineyard, at about 24.7 °C.

Additionally, Table 1 compares the net light intensity and sunlight duration in different areas. The most net light intensity could be witnessed in Yingchuan, whereas the longest sunlight hours could be found in the Shizuishan wine region with almost 183 h. In the aggregate, both net light intensity and sunlight duration in the north were higher than in the south, and the lowest two parameters could be seen in the Hongsipu region, with 15,922,102 J/m^2^/d and 176 h.

#### 3.1.2. Analysis of Soil Nutrient Indicators

The grape root system generally absorbs nutrients from the soil at a depth of 0–60 cm. Nitrogen in soil is beneficial for the growth of wine trees and the accumulation of aromatic substances, and available potassium and phosphorus are favorable for the growth of fruits [24]. Therefore, we analyzed soil pH values and three elements, including available nitrogen, available potassium, and available phosphorus, in these ten vineyards. Several reports have shown that soils with pH values between 6.0 and 7.5 are ideal for the growth of wine grapes [1,25], while excessive acidic and alkaline soil conditions are detrimental to its growth. The pH value results were shown in Table 1, ranging from 7.97 in HD1 to 8.43 in LHY, and the differences between the two locations in the same wine region were less than 0.2. However, in the Yongning wine region, the pH values showed more differences between the two vineyards (8.08 in LL and 8.37 in XXW). These data represented that the pH in southern areas, including Hongsipu and Qingtongxia, was higher than that in Shizuishan and Yinchuan.

**Figure 1:**
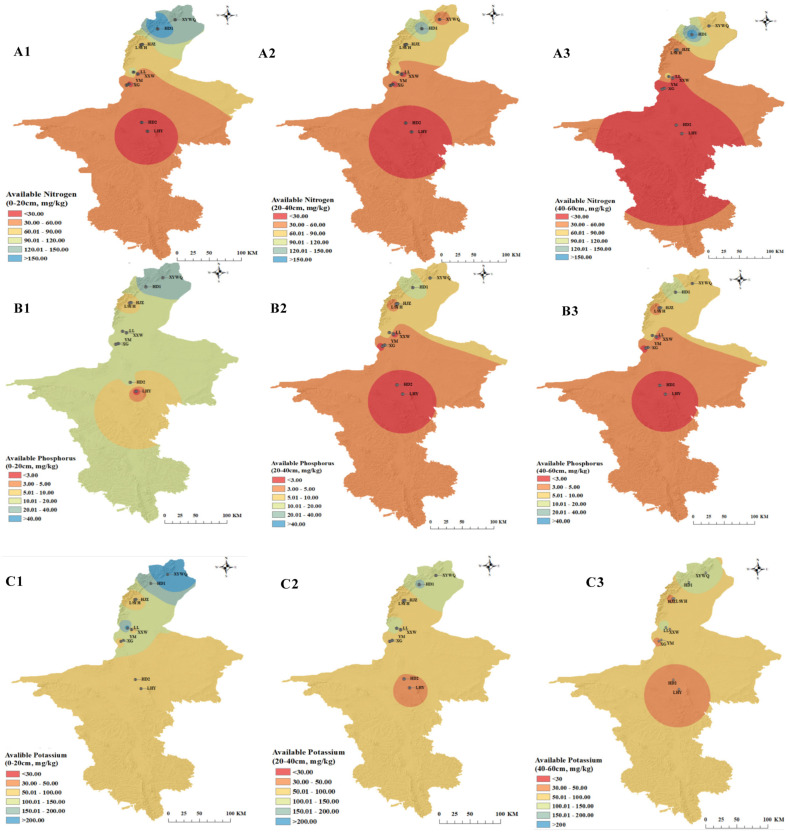
Soil available nitrogen (**A1**–**A3**), soil available potassium (**B1**–**B3**), and soil available phosphorus (**C1**–**C3**) in ten vineyards, including YWQ (Xiyuwangquan), HD1 (Hedong), LSYH (Lanshanyunhao), HJZ (Heijinzun), LL (Lilan), XXW (Xixiawang), YM (Yuma), XG (Xige), LHY (Longhuiyuan), and HD2 (Huida).

In order to analyze the standards of nutrients better, soil nutrient classification standards have been developed (Table S1). As shown in Figure 1(A1–A3), acute deficiencies were identified in all three depths of soil in the Hongsipu region. In contrast, Shizuishan HD1 had the highest fast-acting N content of all vineyards, ranging from 142.21 at 20–40 cm to 215.63 mg/kg at 0–20 cm. However, in another vineyard belonging to the same region, XYWQ was classified with the second level at 0–20 cm, the fifth level at 40–60 cm, and the fourth level at 40–60 cm. In the rest of the wine regions, the content of this element was deficient except at all depths except at 0–20 and 20–40 cm in LL. The Hongsipu producing area had lower available nitrogen content, whereas the higher content of available nitrogen in the Shizuishan production area may be due to the higher alkalinity of the soil in the south than in the Tokoyama area [26].

With respect to available phosphorus (Figure 1(B1–B3)), abundant conditions could be witnessed at 0–20 cm in XYWQ, 0–20 and 20–40 cm in HD1, and 0–20 cm in LL, whereas deficient and below standard conditions could be found in the most of the areas at the soil of deeper than 20 cm, ranging from the lowest content of 1.04 mg/kg at 20–40 cm in LHY to 9.91 mg/kg at 20–40 cm in LL. A possible explanation for this was that in alkaline soils, most of the phosphorus was immobilized, reducing the amount of effective phosphorus that can be used directly by the wine grapes [27,28].

In all five production areas, the differences in available potassium (Figure 1(C1–C3)) were illustrated as follows: The available potassium content decreased as the soil depths were lowered. Hongsipu still had the lowest abundance. In contrast, the Shizuishan and the LL vineyard had higher contents (above 150 mg/kg in three depths of soil in LL). This content in the whole region was classified above the acute deficient standard, implying that this enabled adequate preparation of the grapes for absorbing potassium during ripening.

The content of the organic matrix (Table 2) in all regions except the HD1 vineyard basically showed a deficient state. The contents in HD1 at all depths were nearly 45 g/kg, representing the highest average level. Meanwhile, the organic matrix was concentrated at 0–20 cm of soil, while LHY had a higher content (46.53 g/kg) at 40–60 cm of soil. All of the contents examined were above 3 g/kg, indicating that there were adequate nutrients that could be used by the wine grapes.

### 3.2. Analysis of All Grape Indicators in the Wine Regions and Vineyards

**Figure 2:**
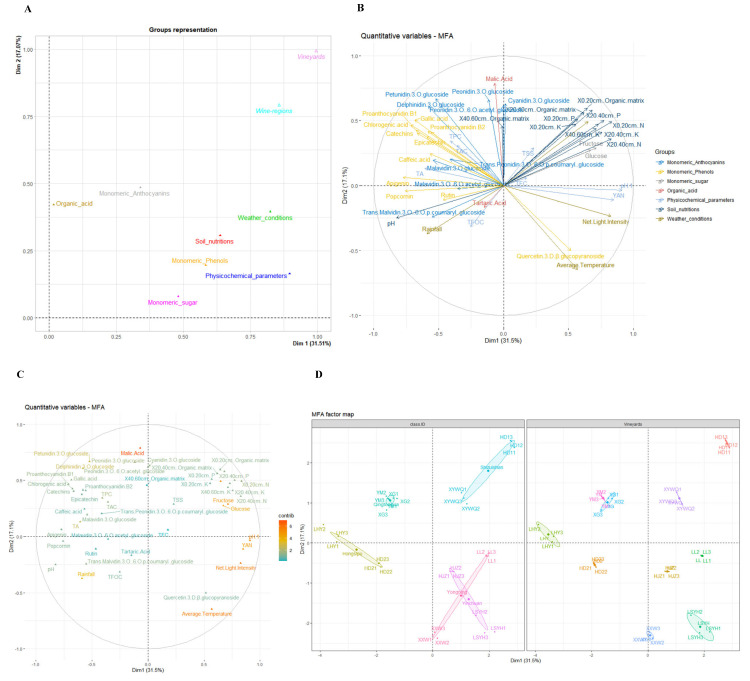
Multiple factor analysis (MFA) of grapes. The correlation between groups and dimensions. Wine regions and vineyards were supplementary groups, while soil nutrients, weather conditions, physiochemical parameters, monomeric sugars, organic acids, monomeric phenols, and monomeric anthocyanins were active groups (**A**). Correlation between quantitative variables and dimensions (**B**). The contribution of quantitative variables to the definition of the dimensions (**C**). Relationships between wine regions (vineyards) and relationships between wine regions (vineyards) and variables (**D**).

This study measured ripeness indicators, phenolic compounds, and monomeric substances, which were shown by multiple factor analysis (MFA) diagrams. MFA takes into account the contribution of all active groups of variables to define the distance between individuals [29]. Figure 2A was used to extract the results for groups of variables. The first two dimensions of group representation describe nearly 49% of the variation. We classified wine regions and vineyards as supplementary groups and regarded the rest of the groups as active groups. The first dimension explained 31.51% of the variation, and the second dimension explained 17.07%. Groups of weather conditions, soil nutrition, monomeric phenols, and physicochemical parameters had a positive and higher contribution to dimension 1, whereas organic acid and monomeric anthocyanins had a higher contribution to dimension 2 than the groups mentioned.

From the perspective of all elements, Figure 2B illustrates the correlation between quantitative variables and dimensions, and Figure 2C highlights the contributing quantitative variables. It was noticeable that all of the soil nutrients, except the 40–60 cm organic matrix and soil pH, had a strong positive relationship with both dimension 1 and dimension 2. These soil nutrients also had a high positive correlation with each other, but they had a completely negative correlation with soil pH, which was negatively related to dimension 1. Weather conditions, sunlight hours, net light intensity, and the average temperature had the same positive correlation with dimension 1, whereas rainfall was negatively related to both dimension 1 and the three weather indicators. It was remarkable that all weather indicators had high contribution values to the two dimensions. Consistent with prior research [30], a high content of available nitrogen in soil could result in a high concentration of TSS, and a high soil pH value was positively correlated with TAC. In contrast, the pH of grapes had a considerable correlation with dim1, followed by yeast assimilable nitrogen (YAN) and titratable acid (TA). Additionally, a high content of available nitrogen could also result in a high level of these three substances [31,32]. According to a previous study [33], the pH of grapes is negatively related to soil pH.

Monomeric sugars had a high correlation and contribution to dim1. With respect to organic acids, malic acid was the strongest and the most positive factor correlating with dim2, but tartaric acid seemed not to have many contributions to the dimensions. Basically, monomeric anthocyanins and phenols almost all had a negative relationship with dim1 but a positive relationship with dim2. Among these substances, petunidin-3-*O*-glucoside and delphinidin-3-*O*-glucoside had higher contributions. However, the contents of these two monomeric anthocyanins were a small part of the total anthocyanin content. As a result, they could not significantly contribute to the TAC and made it correlate less with the two dimensions.

Figure 2D illustrates the MFA factor map for wine regions and vineyards. Individuals with similar profiles were gathered together on the factor maps. As described in the previous paragraph, the first dimension represented a high correlation with sunlight hours, monomeric sugars, grape pH, YAN, and some of the soil nutrients; the second dimension represented a high concentration of malic acid. Therefore, Shizuishan distribution in quadrant 1 was evaluated as having a higher content of monomeric sugars (ranging from 131.75 to 136.67 g/L of glucose and 131.44 to 139.40 g/L of fructose), a higher content of YAN (ranging from 180.5 to 235.5 mg/L), and a higher content of malic acid (ranging from 2.47 to 3.75 mg/L). Such results could be due to longer sunlight hours (180.95 h) and abundantly available nitrogen in the soil (Table 3), aligning with previous studies [27,34]. Additionally, soil nutrition categories in this region were mostly recognized as medium-level or above. Several studies have illustrated that high potassium levels can increase the pH values of must and wine [35,36]; thus, abundant potassium content in Shizuishan could explain its higher pH value in grapes. Moreover, in this region, HD1 had better performance than XYWQ in the parameters mentioned above.

The Hongsipu wine region gathered around the negative coordinates of dim1, indicating that it had high apigenin and myricetin content (ranging from 5.09 to 5.83 mg/L of apigenin and from 24.07 to 24.72 mg/L of myricetin, respectively) and high malvidin-3-*O*-(6-*O*-acetyl)-glucoside content (ranging from 5.14 to 9.43 mg/g). Because of its southern location, the Hongsipu region had lower ripeness levels than the other regions, which were demonstrated by the high concentration of TA (ranging from 5.15 in HD2 to 5.26 g/L in LHY) and lower levels of reducing substances and phenolic compounds, especially anthocyanin.

It was noticeable that Qingtongxia had an excellent separation from the others. It showed higher contents of most of the monomeric anthocyanins and phenols. In contrast, Yongning and Yinchuan were both located in quadrant 4. and each of the vineyards belonging to the corresponding wine region had different relationships with different dimensions; as a result, it was better to analyze them separately. As shown in the MFA factor map for vineyards, compared to HJZ, LL and LSYH (positive coordinate of dim1) had higher pH values in grapes and a higher content of YAN, which could be explained by its high concentration of available potassium and nitrogen. XXW and LSYH had a strong negative correlation with dim2; as a result, they had higher concentrations of quercetin-3-D-β-glucopyranoside. However, LSYH had a more positive correlation with dim1 than XXW, and the average temperature was one of the contributing factors, indicating that LSYH performed better for the factors highly related to dim1, including YAN, grape pH, etc.

### 3.3. Oenology and Physicochemical Indicators of Wine

Studies have shown that higher sugar content can stimulate yeast to produce more alcohol [37]. The alcohol content of Qingtongxia wines in this study differed significantly (*p* < 0.05) from that of other production areas. This could be attributed to the higher soluble solid content of the raw grapes, which differed significantly (*p* < 0.05) from that of other production areas. In contrast, the alcohol content was significantly lower (*p* < 0.05) in the wines from the Hongsipu region. However, high alcohol content tends to weaken the fruity and floral aromas of the wines [38]. Combined with the analysis of aroma data (Table 4), we found that most of the esters that produce more aroma, such as ethyl acetate, ethyl butyrate, and isoamyl acetate, were not detected in the wines from these two appellations. In addition, the glycerol content was higher in the Shizuishan appellation. And relevant studies have shown that glycerol is closely related to sugar content [39]. Therefore, the glycerol content was higher in the Shizuishan wines.

Notably, wines from Hongsipu exhibited significantly lower pH levels compared to those from Yinchuan and Yongning (*p* < 0.05), a pattern that mirrored the pH characteristics observed in their respective grape sources. Furthermore, elevated lactic acid concentrations—attributable to fermentation processes—were consistently detected in wines from Yinchuan and Yongning. This biochemical profile collectively contributed to a softer sensory profile in wines originating from these two regions.

Phenolic compounds have an important role in the health benefits, stability, and color of the wine [40]. The wines from Yinchuan had higher TPC (from 344.24 to 394.28 mg/L), TFC (from 1978.30 to 2022.24 mg/L), and TFOC (from 322.03 to 315.42 mg/L) content, showing significant differences (*p* < 0.05) when compared to the other production areas. The highest TAC content was found in wines from the Yongning appellation and differed significantly (*p* < 0.05) from other appellations. Previous studies have illustrated that light and temperature can significantly impact the accumulation of anthocyanin content [41,42], Hongsipu, which had unfavorable weather and soil nutrients, had the lowest content of TAC.

### 3.4. Analysis of Wine Indicators

**Figure 3:**
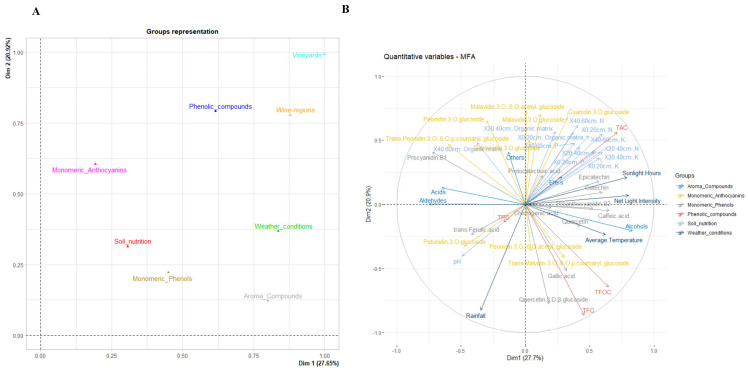

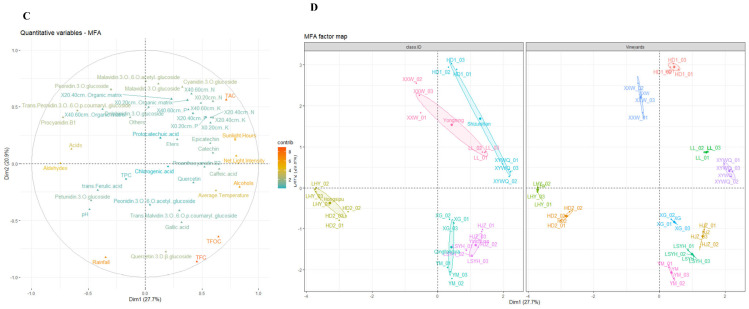
Multiple factor analysis (MFA) of wines. The correlation between groups and dimensions. Wine regions and vineyards were supplementary groups, while soil nutrients, weather conditions, phenolic compounds, monomeric phenols, monomeric anthocyanins, and volatile compounds were active groups (**A**). Correlation between quantitative variables and dimensions (**B**). The contribution of quantitative variables to the definition of the dimensions (**C**). Relationships between wine regions (vineyards) and relationships between wine regions (vineyards) and variables (**D**).

In this study, wine phenolics, volatile compounds, and monomeric phenols were measured and analyzed via MFA (Figure 3A). The results showed that the first two dims described almost 49% of the variables. In this study, wine regions and vineyards were categorized as complementary groups, and the remaining groups were considered as active groups. The first dim explained 27.65% of the variance, and the second dim explained 20.92% of the variance. Volatile compounds, phenolics, and weather conditions contributed more to dim 1, while phenolics and monomeric anthocyanins contributed more to dim 2. To further analyze the relationships between all variations, Figure 2B illustrates the correlations between quantitative variables and the dims, and Figure 2C highlights the influencing quantitative variables. Most of the soil nutrients had strong positive correlations with dim 1 and dim 2 and contributed more to dim 1 and dim 2, except for soil PH. Regarding climatic conditions, sunshine hours, mean temperature, and net photosynthetic intensity were positively correlated with dim 1 and contributed more to dim 1. Regarding phenolics, quercetin-3-D-β-glucoside and gallic acid in wines were negatively correlated with dim 2. TAC was positively correlated with both dim 1 and dim 2, whereas TPC, TFC, and TFOC were negatively correlated with dim 2. For wine monomeric anthocyanins, most of them were positively correlated with dim 2, with higher contributions from cyanidin-3-*O*-glucoside, peonidin-3-*O*-glucoside, malvidin-3-*O*-glucoside, and malvidin-3-*O*-(6-*O*-acetyl)-glucoside. In addition, for volatiles, alcohols were positively correlated with dim 1, whereas aldehydes and acids were negatively correlated with dim 1, and other volatiles and esters were only related to dim 2, similar to previous studies [24].

Subsequently, two maps revealed the separation of the different wine regions and vineyards between the two dimensions (Figure 3D). It was clear that wines made from Yinchuan, Qingtongxia, and Hongsipu gathered closely on the map; however, wines made from Shizuishan and Yongning seemed to be the opposite. Hongsipu was distributed on the west of the map; therefore, it had high contents of acids (3631.14 μg/L in LHY and 6560.81 μg/L in HD2) and aldehydes (1717.12 μg/L in LHY and 537.77 μg/L in HD2). Both Qingtongxia and Yinchuan are located in quadrant 4, implying that they had higher contents of peonidin 3-*O*-(6-*O*-acetyl)-glucoside, trans-Malvidin 3-*O*-(6-*O*-p-coumaryl)-glucoside, quercetin-3-D-β-glucoside, and gallic acid. When searching for a vineyard that was highly and positively correlated with dim1, XYWQ was selected. Thus, the contents of epicatechin, PB2, epicatechin, and catechin in XYWQ were higher than the others, which could be explained by the abundant contents of N, K, and P at 0–20 cm soil. Additionally, some studies found that sunlight could be a factor impacting the content of monomeric phenols [43]; therefore, considering the net light intensity and sunlight hours in this vineyard were at a high level, wine made from XYWQ had high contents of these substances. HD1, belonging to the same wine region, was positively correlated with both dimensions, and its wine had higher contents of malvidin 3-*O*-glucoside (90.55 mg/L) and malvidin 3-*O*-(6-*O*-acetyl)-glucoside (37.63 mg/L). This phenomenon might positively relate to organic matrices in soil, and the level of nutrients in HD1 was classified as high. Moreover, up to 183 h of sunlight could explain the excellent concentrations of these substances in the Shizuishan wine region. Similarly, due to the high correlation of dim2, XXW also had excellent concentrations of most of the monomeric anthocyanins.

With regard to volatile compounds, a total of 31 volatile compounds were detected in wines from 10 vineyards in five appellations. Table 4 lists the 31 volatile compounds detected in the wines. The thresholds and descriptions of these aromas were obtained from data in the literature [44,45,46]. Among them, alcohols and esters were more diverse and concentrated, while fatty acids, aldehydes, and ketones were less abundant as minor compounds. In this study, the total volatile compound concentrations of the five production areas ranged from 153.43 mg/L to 223.42 mg/L. Among them, wines from the Yinchuan production area, HZJ, had the highest aroma content, and wines from the Hongshibao production area, LHY, had the lowest aroma content. Studies have shown that the aroma of wine depends on factors such as the grape variety, climatic conditions, cultural practices, and fermentation processes [47]. In the present study, the grape variety, cultivation practices, and fermentation conditions used were the same for all wines. Therefore, the differences in volatile compounds between wines may be due to factors such as climatic conditions and soils in different production areas. This suggests that the volatile compounds of wines are influenced by the terroir of different production areas [48,49,50]. Alcohols were the most abundant and concentrated category in wines from different regions. The total alcohol concentrations in the wines ranged from 122.32 mg/L to 177.95 mg/L. Among them, the Yongning LL wines had the highest alcohol concentration, which was significantly different from the other wines (*p* < 0.05) and mainly consisted of isoamyl alcohol, phenylethanol, hexanol, isobutanol, and 1-butanol. The same was true for the wines from the other appellations, indicating that these alcohols contribute excellently to the wine aroma in wines from different appellations.

Esters are one of the most important classes of compounds in wine and are produced mainly by enzymatic reactions during yeast fermentation or during the ethanolysis of acetyl coenzyme A formed during fatty acid synthesis or degradation [44]. In this study, a total of seven esters were detected in wines from each appellation. These esters contributed positively to the overall quality of the wines, most of them imparting a fruity aroma to the grapes [50]. Among these esters, ethyl acetate and ethyl caprylate are the most abundant. The concentration of ethyl acetate in each wine was classified in the following order: LL > XYWQ > LHY > XG > HD1 > YM > HD2 > HJZ > XXW > LSYH. The concentration of ethyl caprylate in each wine was ranked in the following order: HJZ > XYWQ > LL > HD1 > HD2 > XG > LHY > XXW > YM > LSYH. The concentrations of ethyl acetate and ethyl caprylate in Cabernet Sauvignon wines ranged from 4.64 to 12.74 and from 3.15 to 31.31, respectively. However, there were significant variations in the concentrations of ethyl acetate and ethyl caprylate in wines from different regions (*p* < 0.05), which may be related to factors such as sunshine duration, climatic conditions, and soil conditions in different wine regions [51].

Fatty acid synthesis depends on the composition of the must and the fermentation conditions [52]. In this study, a total of five fatty acids were detected in all wines. The highest octanoic acid content was found in HD2 wines from Hongsipu, with a significant difference (*p* < 0.05) compared to other production areas. The lowest content was found in HJZ wines from Yinchuan, with a significant difference (*p* < 0.05) from other production areas. Caprylic acid is a product of the fatty acid metabolism of yeast, and its content is closely related to the type of yeast and the final stage of fermentation [53]. On the other hand, isobutyric and capric acids are not related to wine quality but are related to the complexity of wine aroma. An appropriate level of fatty acids in wine is necessary to enhance the complexity of the wine aroma [44,45]. At a concentration of 4–10 mg/L, fatty acids can give wines a mild aroma, whereas at a concentration of more than 20 mg/L, fatty acids can reduce the quality of the wine aroma [44]. In terms of aldehydes and other compounds, Château Rouge wines contain relatively high levels, with the LHY acetaldehyde content of the Château Rouge appellation being significantly higher than that of the other appellations (*p* < 0.05). Acetaldehyde is mainly produced by the metabolic activity of yeasts and lactic acid bacteria [54]. The acetaldehyde content of wines varies considerably from one region to another, depending on the composition of the microbial community, the maturity of the grapes, the fermentation process, and other factors [55,56].

Overall, there were differences in volatile compounds in wines from different production areas. The higher alcohol content of Yongning and Yinchuan wines may give the wines a more floral aroma. The higher content of esters in Shizuishan wines may give the wines a more fruity and creamy aroma.

## 4. Conclusions

This study systematically reveals the regulatory mechanisms of terroir elements on grape and wine quality in wine-producing areas of Ningxia. The results from north to south showed that the gradual decline in effective soil nutrients and heat accumulation led to a synchronous decline in fruit ripening parameters. The Shizuishan appellation achieved an optimal glucose accumulation due to superior heat conditions and soil nitrogen levels. In Hongshibao, climatic adversity during the harvest period inhibited the synthesis of phenolic substances, especially anthocyanins, resulting in poor wine color presentation. The Qingtongxia appellation became a phenolic enrichment area with an outstanding total amount of monomeric phenols, while Yongning showed a varietal-specific anthocyanin enrichment phenomenon. There are significant differences in the terroir of the different vineyards in Yinchuan: The volatile ester profile in HJZ is typical of fruity aromas; the XXW vineyard has a higher content of quercetin-3-D-β-glucopyranoside and most of the monomeric anthocyanins in the grapes, which enhances the complexity of the phenolic composition; the LSYH and LL vineyards have more available nitrogen (YAN) and must pH characteristics, resulting in a higher proportion of alcoholic volatiles in the wine. The LSYH and LL vineyards are characterized by a higher available nitrogen (YAN) and a higher pH of the must, resulting in more volatile alcohols.

## Figures and Tables

**Table 1:** Climate conditions and soil pH value in five wine regions (ten vineyards).

Wine Regions	Vineyards	Average Temperature (°C)	Average Humidity (%)	Rainfall (mm)	Net Light Intensity (Net, J/m^2^/d)	Sunlight Hours (h)	pH
Shizuishan	XYWQ	26.1	44.8	24.77	17,000,185.37	182.95	8.20
	HD1	25.1	52.9				7.97
Yinchuan	LSYH	27.4	44.9	33.09	17,482,354.61	179.64	8.16
	HJZ	26.5	44.5				8.23
Yongning	LL	26.5	46.8	28.22	16,831,369.98	178.75	8.08
	XXW	26.2	48.6				8.37
Qingtongxia	YM	25.4	51.7	32.92	16,434,452.67	180.85	8.42
	XG	25.8	47.4				8.35
Hongsipu	LHY	24.7	46.0	32.17	15,922,102	176	8.43
	HD2	25.5	46.7				8.35

**Table 2:** Soil organic matter content (g/kg) in different wine-producing regions.

	Shizuishan		Yinchuan		Yongning		Qingtongxia		Hongsipu	
	XYWQ	HD1	LSYH	HJZ	LL	XXW	YM	XG	LHY	HD2
0~20 cm	30.85 ± 0.25 b	44.96 ± 0.39 a	11.64 ± 0.19 c	6.98 ± 0.03 f	9.86 ± 0.24 d	4.73 ± 0.09 g	6.87 ± 0.07 f	4.45 ± 0.03 g	2.71 ± 0.02 h	9.02 ± 0.01 e
20~40 cm	9.02 ± 0.01 d	45.91 ± 0.08 a	10.58 ± 0.16 b	7.7 ± 0.16 e	9.78 ± 0.11 c	2.95 ± 0.04 j	6.82 ± 0.1 f	4.19 ± 0.02 h	3.3 ± 0.01 i	5.19 ± 0.03 g
40~60 cm	13.13 ± 0.18 c	44.88 ± 0.44 b	9.19 ± 0.14 d	6.34 ± 0.02 e	8.9 ± 0.25 d	4.86 ± 0.08 f	4.05 ± 0.11 f	2.8 ± 0.01 g	46.53 ± 0.85 a	4.22 ± 0 f

Note: Different letters in the row indicate significant differences (Duncan test, *p* < 0.05) among treatments.

**Table 3:** Basic indicators of grapes and wines in five wine regions (ten vineyards).

	Shizuishan		Yinchuan		Yongning		Qingtongxia		Hongsipu	
	XYWQ	HD1	LSYH	HJZ	LL	XXW	YM	XG	LHY	HD2
Grapes										
TA (g/L)	3.25 ± 0.00 g	4.57 ± 0.01 c	3.08 ± 0.00 h	3.59 ± 0.00 f	3.14 ± 0.01 h	4.39 ± 0.00 d	3.87 ± 0.03 e	3.77 ± 0.04 e	5.26 ± 0.02 a	5.15 ± 0.02 b
TSS (g/L)	25.16 ± 0.03 e	25.70 ± 0.01 d	27.61 ± 0.01 c	28.03 ± 0.02 b	28.07 ± 0.01 b	19.56 ± 0.00 g	27.62 ± 0.01 c	28.64 ± 0.01 a	22.58 ± 0.03 f	25.68 ± 0.01 d
pH	3.74 ± 0.01 c	3.81 ± 0.01 a	3.76 ± 0.01 bc	3.64 ± 0.00 d	3.79 ± 0.01 ab	3.74 ± 0.01 c	3.62 ± 0.01 de	3.59 ± 0.00 e	3.50 ± 0.00 g	3.55 ± 0.00 f
YAN (mg/L)	180.50 ± 1.50 de	235.50 ± 0.50 a	215.50 ± 1.50 b	200.00 ± 3.00 c	230.50 ± 2.50 a	200.00 ± 3.00 c	174.00 ± 4.00 ef	169.50 ± 2.50 ef	167.50 ± 1.50 f	189.50 ± 0.50 cd
TPC (mg/g)	52.02 ± 6.01 ab	46.64 ± 4.72 b	52.91 ± 3.50 ab	43.09 ± 3.62 b	46.09 ± 1.90 b	45.12 ± 2.28 b	60.41 ± 6.80 a	60.16 ± 3.72 a	52.02 ± 5.85 ab	47.07 ± 5.65 b
TFC (mg/g)	38.92 ± 0.76 b	42.39 ± 0.19 a	35.25 ± 3.63 cd	33.30 ± 1.34 d	28.26 ± 0.14 e	39.80 ± 2.20 ab	33.53 ± 1.21 d	25.85 ± 0.42 e	37.81 ± 0.34 bc	35.33 ± 0.30 cd
TFOC (mg/g)	11.99 ± 0.06 f	12.02 ± 0.08 f	13.44 ± 0.00 d	13.81 ± 0.07 c	10.92 ± 0.04 h	13.06 ± 0.04 e	13.96 ± 0.02 b	11.36 ± 0.11 g	12.10 ± 0.00 f	14.22 ± 0.00 a
TAC (mg/g)	29.99 ± 0.11 b	23.62 ± 0.02 cd	24.00 ± 0.20 cd	22.50 ± 0.18 de	20.96 ± 0.16 e	22.60 ± 0.04 de	25.20 ± 1.29 c	25.43 ± 0.17 c	34.33 ± 2.12 a	17.99 ± 0.65 f
Wine										
Alcoholic degree (%)	14.75 ± 0.04 f	14.88 ± 0.34 e	15.18 ± 0.01 d	16.52 ± 0.04 a	15.53 ± 0.08 c	12.74 ± 0.01 h	15.47 ± 0.02 c	15.76 ± 0.00 b	14.87 ± 0.02 e	13.19 ± 0.00 g
Reducing substances (g/L)	2.65 ± 0.07 c	2.77 ± 0.06 a	2.54 ± 0.06 de	2.41 ± 0.01 g	2.41 ± 0.01 g	2.51 ± 0.01 ef	2.37 ± 0.01 g	2.72 ± 0.01 b	2.57 ± 0.01 d	2.47 ± 0.01 f
pH	3.60 ± 0.00 c	3.44 ± 0.04 f	3.69 ± 0.00 a	3.60 ± 0.01 c	3.62 ± 0.01 b	3.45 ± 0.00 f	3.58 ± 0.00 d	3.62 ± 0.01 b	3.37 ± 0.01 g	3.51 ± 0.00 e
Glycerol (g/L)	8.85 ± 0.15 b	9.45 ± 0.55 a	9.13 ± 0.05 a	9.55 ± 0.05 a	9.55 ± 0.15 a	8.65 ± 0.15 b	9.30 ± 0.10 a	8.60 ± 0.10 b	7.95 ± 0.15 c	8.85 ± 0.25 b
TPC (mg/L)	365.54 ± 2.21 b	335.26 ± 2.27 d	344.24 ± 1.31 c	394.28 ± 5.94 a	243.39 ± 1.81 g	307.8 ± 4.19 f	315.24 ± 0.63 e	239.54 ± 0.73 g	360.92 ± 2.02 b	347.32 ± 0.96 c
TFC (mg/L)	1650.81 ± 25.97 c	1177.97 ± 18.91 f	1978.3 ± 14.29 b	2022.24 ± 4.68 ab	1666.96 ± 8.10 c	1265.83 ± 20.39 e	2048.98 ± 13.51 a	1708.98 ± 11.77 c	1311.67 ± 29.22 e	1590.55 ± 16.43 d
TFOC (mg/L)	317.11 ± 6.97 b	277.16 ± 1.02 d	315.42 ± 4.28 b	322.03 ± 11.04 ab	323.47 ± 0.38 ab	288.44 ± 0.94 c	328.80 ± 1.00 a	317.42 ± 3.45 b	277.77 ± 1.39 d	297.06 ± 2.36 c
TAC (mg/L)	161.53 ± 3.76 c	162.97 ± 2.41 c	147.32 ± 0.17 d	150.40 ± 5.09 d	176.84 ± 4.90 b	180.94 ± 8.02 a	135.71 ± 1.25 e	147.58 ± 0.17 d	108.92 ± 1.75 f	112.33 ± 3.91 f

Note: Different letters in the row indicate significant differences (Duncan test, *p* < 0.05) among treatments.

**Table 4:** Volatile compounds in five wine regions (ten vineyards).

**Compounds**	**Threshold (mg/L)**	Odor Descriptor	Shizuishan		Yinchuan		Yongning		Qingtongxia		Hongsipu	
			XYWQ	HD1	LSYH	HJZ	LL	XXW	YM	XG	LHY	HD2
Alcohols												
Isobutanol	1.11	fusel, alcohol A	3.97 ± 0.06 d	4.41 ± 0.14 c	4.79 ± 0.02 b	6.28 ± 0.16 a	4.77 ± 0.23 b	4.01 ± 0.02 d	4.87 ± 0.17 b	4.73 ± 0.08 b	2.93 ± 0.07 e	3.72 ± 0.29 d
Isoamyl alcohol	1.22	cheese, solvent B	122.60 ± 1.11 d	111.70 ± 5.90 e	121.52 ± 0.88 d	150.48 ± 0.03 a	130.62 ± 0.95 bc	111.79 ± 0.20 e	128.81 ± 0.23 c	134.58 ± 3.58 bc	96.04 ± 0.36 f	107.94 ± 2.50 e
4-Methyl-1-pentanol	-	-	0.09 ± 0.00 a	0.09 ± 0.00 a	0.06 ± 0.00 e	0.08 ± 0.00 c	0.06 ± 0.00 d	0.04 ± 0.00 g	0.05 ± 0.00 f	0.05 ± 0.00 f	0.05 ± 0.00 f	0.08 ± 0.00 b
3-methyl-1-pentanol	1.35	vinous, herbaceous	0.25 ± 0.03 c	0.26 ± 0.02 bc	0.25 ± 0.01 c	0.29 ± 0.01 b	0.26 ± 0.01 c	0.20 ± 0.00 d	0.26 ± 0.01 c	0.26 ± 0.00 bc	0.32 ± 0.01 a	0.29 ± 0.01 b
1-Hexanol	8.00	green, grass	3.36 ± 0.18 c	4.31 ± 0.04 b	3.57 ± 0.02 c	2.70 ± 0.09 d	4.52 ± 0.20 b	4.96 ± 0.04 a	2.89 ± 0.05 d	3.39 ± 0.18 c	3.40 ± 0.07 c	2.93 ± 0.09 d
3-Hexen-1-ol	0.40	Green, floral	0.05 ± 0.00 c	0.09 ± 0.00 a	0.08 ± 0.00 b	0.03 ± 0.00 ef	0.04 ± 0.00 e	0.04 ± 0.00 d	0.03 ± 0.00 g	0.00 ± 0.00 h	0.03 ± 0.00 f	0.02 ± 0.00 g
(R,R)-2,3-Butanediol	1.62	Rubber G	0.56 ± 0.01 b	0.45 ± 0.01 c	0.77 ± 0.01 a	0.57 ± 0.03 b	0.00 ± 0.00 d	0.00 ± 0.00 d	0.00 ± 0.00 d	0.00 ± 0.00 d	0.00 ± 0.00 d	0.00 ± 0.00 d
1-Nonanol	0.60	Green	0.16 ± 0.00 c	0.12 ± 0.01 e	0.10 ± 0.00 f	0.05 ± 0.00 h	0.19 ± 0.01 b	0.27 ± 0.00 a	0.14 ± 0.00 d	0.12 ± 0.00 e	0.13 ± 0.01 e	0.07 ± 0.00 g
Phenethyl alcohol	-	-	33.30 ± 0.37 b	22.56 ± 1.35 de	22.95 ± 0.52 d	15.55 ± 0.31 g	37.06 ± 0.03 a	28.81 ± 0.12 c	22.25 ± 0.56 de	33.19 ± 0.41 b	19.10 ± 0.09 f	21.58 ± 0.24 e
Heptaethylene glycol	-	-	0.09 ± 0.00 a	0.00 ± 0.00 ef	0.01 ± 0.00 de	0.01 ± 0.00 cd	0.00 ± 0.00 f	0.02 ± 0.00 b	0.00 ± 0.00 f	0.00 ± 0.00 f	0.00 ± 0.00 f	0.01 ± 0.00 c
1-Butanol	150.00	Medicinal, alcohol	0.28 ± 0.00 cd	0.16 ± 0.03 f	0.44 ± 0.02 b	0.57 ± 0.02 a	0.30 ± 0.01 c	0.20 ± 0.00 e	0.26 ± 0.01 d	0.31 ± 0.01 c	0.16 ± 0.01 f	0.16 ± 0.00 f
1-Octanol	0.01	intense citrus, roses	0.13 ± 0.00 d	0.18 ± 0.00 a	0.13 ± 0.00 d	0.05 ± 0.00 f	0.13 ± 0.00 d	0.15 ± 0.00 c	0.06 ± 0.00 f	0.10 ± 0.00 e	0.17 ± 0.02 ab	0.16 ± 0.00 bc
Subtotal			164.84 ± 1.62 b	144.32 ± 7.48 e	154.66 ± 0.36 cd	176.67 ± 0.03 a	177.95 ± 1.44 a	150.50 ± 0.13 d	159.62 ± 1.04 bc	176.72 ± 3.43 a	122.32 ± 0.47 g	136.97 ± 2.44 f
Esters												
Ethyl Acetate	7.50	pineapple, fruity, balsamic	11.63 ± 1.01 b	9.78 ± 0.28 c	4.64 ± 0.07 e	8.86 ± 0.31 cd	12.74 ± 0.83 a	8.52 ± 0.25 d	9.48 ± 0.30 cd	11.43 ± 0.64 b	11.59 ± 0.38 b	8.95 ± 0.13 cd
Isoamyl acetate	0.003	banana, fruity, sweet	1.11 ± 0.17 d	1.87 ± 0.04 a	0.40 ± 0.04 h	0.97 ± 0.00 de	1.72 ± 0.12 b	0.62 ± 0.02 g	1.04 ± 0.05 de	1.36 ± 0.03 c	0.77 ± 0.02 fg	0.90 ± 0.02 ef
Hexyl acetate	1.29	apple, cherry, pear, floral B	0.12 ± 0.00 b	0.12 ± 0.01 b	0.00 ± 0.00 f	0.06 ± 0.00 d	0.13 ± 0.00 a	0.07 ± 0.00 c	0.07 ± 0.00 c	0.03 ± 0.00 e	0.00 ± 0.00 f	0.00 ± 0.00 f
Ethyl lactate	1.37	lactic, raspberry, fruity, buttery B	0.00 ± 0.00 e	0.00 ± 0.00 e	0.00 ± 0.00 e	0.02 ± 0.00 d	0.00 ± 0.00 e	0.08 ± 0.00 c	0.00 ± 0.00 e	0.00 ± 0.00 e	0.13 ± 0.00 a	0.10 ± 0.00 b
Ethyl octanoate	1.45	pineapple, pear, floral, fruity, brandy B	10.61 ± 0.26 b	6.90 ± 0.08 c	3.15 ± 0.04 i	31.31 ± 0.01 a	6.93 ± 0.06 c	4.08 ± 0.06 g	3.49 ± 0.04 h	5.95 ± 0.01 e	5.46 ± 0.17 f	6.33 ± 0.10 d
Phenethyl acetate	-	-	0.66 ± 0.00 f	0.75 ± 0.02 e	0.67 ± 0.00 f	0.81 ± 0.00 d	1.63 ± 0.03 a	0.80 ± 0.00 d	1.46 ± 0.00 c	1.54 ± 0.02 b	0.61 ± 0.01 g	0.59 ± 0.01 g
Ethyl caprate	-	-	5.26 ± 0.06 a	3.33 ± 0.16 b	0.99 ± 0.01 i	1.21 ± 0.04 h	2.93 ± 0.00 c	1.63 ± 0.01 fg	1.66 ± 0.00 f	2.36 ± 0.03 e	1.52 ± 0.02 g	2.64 ± 0.05 d
Ethyl butyrate	0.02	strawberry, apple, banana	0.69 ± 0.01 a	0.00 ± 0.00 d	0.00 ± 0.00 d	0.00 ± 0.00 d	0.00 ± 0.00 d	0.00 ± 0.00 d	0.00 ± 0.00 d	0.00 ± 0.00 d	0.48 ± 0.01 c	0.53 ± 0.01 b
Ethyl Hexanoate	5	fruity, green apple; floral, violet	2.69 ± 0.00 a	1.55 ± 0.02 b	0.77 ± 0.01 g	0.80 ± 0.01 g	1.42 ± 0.04 c	0.98 ± 0.02 f	0.81 ± 0.00 g	1.06 ± 0.04 e	1.26 ± 0.01 d	1.05 ± 0.02 e
3-Hexen-1-ol	0.40	Green, floral	0.05 ± 0.00 c	0.09 ± 0.00 a	0.08 ± 0.00 b	0.03 ± 0.00 ef	0.04 ± 0.00 e	0.04 ± 0.00 d	0.03 ± 0.00 g	0.00 ± 0.00 h	0.03 ± 0.00 f	0.02 ± 0.00 g
Ethyl nonanoate	1.30	waxy, fruity	0.07 ± 0.00 a	0.03 ± 0.00 b	0.00 ± 0.00 d	0.00 ± 0.00 d	0.00 ± 0.00 d	0.02 ± 0.00 c	0.00 ± 0.00 d	0.00 ± 0.00 d	0.00 ± 0.00 d	0.00 ± 0.00 d
Diethyl succinate	500.00	Light fruity	0.10 ± 0.00 g	0.24 ± 0.01 e	0.15 ± 0.00 f	0.10 ± 0.00 g	0.29 ± 0.01 c	0.29 ± 0.01 c	0.10 ± 0.00 g	0.27 ± 0.00 d	0.32 ± 0.00 b	0.40 ± 0.01 a
Subtotal			32.99 ± 0.97 b	24.66 ± 0.30 d	10.84 ± 0.10 g	44.19 ± 0.36 a	27.81 ± 1.02 c	17.13 ± 0.35 f	18.14 ± 0.39 f	24.00 ± 0.72 d	22.17 ± 0.22 e	21.52 ± 0.17 e
Acids												
Isobutyric acid	200.00	Fatty	0.05 ± 0.00 c	0.05 ± 0.00 c	0.14 ± 0.05 a	0.08 ± 0.00 bc	0.10 ± 0.00 b	0.07 ± 0.00 bc	0.08 ± 0.00 bc	0.08 ± 0.00 bc	0.06 ± 0.00 c	0.08 ± 0.00 bc
Hexanoic acid	3.00	Cheese, rancid, fatty	0.56 ± 0.00 b	0.48 ± 0.01 b	0.00 ± 0.00 c	0.00 ± 0.00 c	0.00 ± 0.00 c	0.00 ± 0.00 c	0.48 ± 0.00 b	0.00 ± 0.00 c	0.00 ± 0.00 c	1.58 ± 0.15 a
Octanoic acid	0.50	rancid, harsh, cheese, fatty acid	2.21 ± 0.01 d	2.69 ± 0.15 c	1.28 ± 0.02 fg	0.41 ± 0.00 i	1.41 ± 0.02 f	1.74 ± 0.01 e	1.17 ± 0.02 gh	1.06 ± 0.04 h	3.23 ± 0.02 b	4.52 ± 0.09 a
Syrene	1.40	resin, floral	0.53 ± 0.01 a	0.00 ± 0.00 b	0.00 ± 0.00 b	0.00 ± 0.00 b	0.00 ± 0.00 b	0.00 ± 0.00 b	0.00 ± 0.00 b	0.00 ± 0.00 b	0.00 ± 0.00 b	0.00 ± 0.00 b
2-methylbutanoic acid	1.66	-	0.00 ± 0.00 i	0.00 ± 0.00 i	0.63 ± 0.01 a	0.40 ± 0.00 f	0.51 ± 0.00 d	0.60 ± 0.00 b	0.46 ± 0.00 e	0.53 ± 0.02 c	0.35 ± 0.00 h	0.38 ± 0.00 g
Subtotal			3.36 ± 0.02 c	3.22 ± 0.15 c	2.05 ± 0.08 e	0.89 ± 0.01 g	2.02 ± 0.02 e	2.40 ± 0.01 d	2.19 ± 0.02 e	1.67 ± 0.06 f	3.63 ± 0.02 b	6.56 ± 0.24 a
Aldehydes												
acetaldehyde	0.71	fruity, pungent, green, grassy, apple F	0.46 ± 0.02 c	0.41 ± 0.00 d	0.45 ± 0.02 c	0.31 ± 0.01 e	0.48 ± 0.02 c	0.56 ± 0.01 b	0.55 ± 0.01 b	0.41 ± 0.02 d	1.72 ± 0.05 a	0.54 ± 0.01 b
2,3-Pentanedione	-	-	2.44 ± 0.02 b	2.51 ± 0.04 b	2.56 ± 0.00 b	1.37 ± 0.03 b	3.46 ± 0.01 b	23.16 ± 15.46 a	3.21 ± 0.26 b	3.48 ± 0.00 b	3.62 ± 0.04 b	3.31 ± 0.02 b
Total			204.10	175.13	170.56	223.42	211.71	193.76	183.71	206.28	153.45	168.90

Note: Different letters in the row indicate significant differences (Duncan test, *p* < 0.05) among treatments.

## Data Availability

The original contributions presented in this study are included in the article/Supplementary Materials. Further inquiries can be directed to the corresponding author.

## Supplementary Materials

The following supporting information can be downloaded at: https://www.mdpi.com/article/10.3390/foods14081409/s1, Table S1. Classification criteria for soil nutrition.
