# Supplementary material for: Effect of Terroir on Phenolic Content and Aroma Properties of Grapes and Wines
Source: Foods. 2025 Apr 18;14(8):1409. doi: 10.3390/foods14081409 (PMC12026802; doi:10.3390/foods14081409)
Supplement: Supplementary file 1 [file foods-14-01409-s001.zip › foods-3559833-supplementary.pdf]

Table S1. Classification criteria for soil nutrition

Rank	Description	Organic matrix (g/kg)	Available nitrogen (mg/kg)	Available potassium (mg/kg)	Available phosphorus (mg/kg)
1	Very abundant	>40.00	>150.00	>40.00	>200.00
2	Abundant	30.00~40.00	120.00~150.00	20.00~40.00	150.00~200.00
3	Medium	20.00~30.00	90.00~120.00	10.00~20.00	100.00~150.00
4	Deficient	10.00~20.00	60.00~90.00	5.00~10.00	50.00~100.00
5	Very deficient	6.00~10.00	30.00~60.00	3.00~5.00	30.00~50.00
6	Acute deficient	<6.00	<30.00	<3.00	<30.00
